# Effects of Intra-Operative Total Intravenous Anaesthesia with Propofol versus Inhalational Anaesthesia with Sevoflurane on Post-Operative Pain in Liver Surgery: A Retrospective Case-Control Study

**DOI:** 10.1371/journal.pone.0149753

**Published:** 2016-02-22

**Authors:** Alfred Chor San Chan, Qiu Qiu, Siu Wai Choi, Stanley Sau Ching Wong, Albert Chi Yan Chan, Michael G Irwin, Chi Wai Cheung

**Affiliations:** 1 Department of Anaesthesiology, Queen Mary Hospital, Hong Kong, China; 2 Laboratory and Clinical Research Institute for Pain, Department of Anaesthesiology, The University of Hong Kong, Hong Kong, China; 3 Department of Surgery, The University of Hong Kong, Hong Kong, China; Imperial College London, Chelsea & Westminster Hospital, UNITED KINGDOM

## Abstract

**Background:**

Patients receiving total intravenous anesthesia (TIVA) with propofol have been shown to experience less postoperative pain. We evaluated the post-operative analgesic effects of propofol compared with sevoflurane maintenance of anesthesia in liver surgery. This study was registered at ClinicalTrials.gov (NCT02179437).

**Methods:**

In this retrospective study, records of patients who underwent liver surgery between 2010 and 2013 were reviewed. Ninety-five patients anesthetized with propofol TIVA were matched with 95 patients anesthetized with sevoflurane. Numeric pain rating scale (NRS) pain scores, postoperative morphine consumption, side effects and patients’ satisfaction with pain relief were evaluated.

**Results:**

The TIVA group reported lower NRS pain scores during coughing on postoperative days 1 and 2 but not 3 (p = 0.0127, p = 0.0472, p = 0.4556 respectively). They also consumed significantly less daily (p = 0.001 on day 1, p = 0.0231 on day 2, p = 0.0004 on day 3), accumulative (p = 0.001 on day 1, p<0.0001 on day 2 and p = 0.0064 on day 3) and total morphine (p = 0.03) when compared with the sevoflurane group. There were no differences in total duration of intravenous patient controlled analgesia (PCA) morphine use and patient satisfaction. No difference was found in reported side effects.

**Conclusion:**

Patients anesthetized with propofol TIVA reported less pain during coughing and consumed less daily, accumulative and total morphine after liver surgery.

## Introduction

Postoperative pain can be severe after liver surgery due to the upper abdominal incision and pain management can be difficult for this group of patients [1]. Epidural analgesia is relatively contraindicated due to impaired postoperative coagulation profiles and, consequently, large doses of strong opioids may be required in order to achieve adequate pain control. As shown in an audit of postoperative intravenous patient-controlled analgesia (PCA), patients who underwent hepatobiliary and pancreatic surgery were found to report moderate to severe pain scores and high morphine consumption [2]. In addition, liver resection impair opioid metabolism, as reflected by higher plasma morphine concentrations in patients after hepatectomy compared with those after colon resection, leading to a higher incidence of side effects such as sedation [3]. Therefore, opioid-sparing multimodal pain treatment is important for fast-track recovery after liver resection [4]. Identification of novel analgesic techniques is of utmost importance to achieve this goal.

Propofol (2,6-diisopropylphenol) is an intravenous anesthetic. Its pharmacokinetic profile makes it very suitable for total intravenous anesthesia (TIVA) and this is a widely used technique in many centers [5, 6]. Its use results in a rapid onset and offset with fewer side effects including postoperative nausea and vomiting, making it particularly favorable in the ambulatory setting [7]. Studies have been conducted to explore possible anti-nociceptive mechanisms of propofol and its potential role as an analgesic clinically. In animal studies, propofol has been shown to directly depress the dorsal horn neurons in the spinal cord [8], inhibit the phosphorylation of N-methyl-D-aspartate receptor NR1 subunit [9], and inhibit the cannabinoid CB1 and CB2 receptors [10]. In human volunteers, hypnotic doses of propofol at 3.5 mcg/ml decreased pain-related regional blood flow to the thalamus and anterior cingulate cortex [11]. Propofol significantly decreased pain scores by 40% and areas of hyperalgesia and allodynia in human volunteers [12]. Work on propofol’s preferential binding to the HCN1 pacemaker channels further reinforce its anti-hyperalgesic effects [13, 14]. Propofol has been shown to be anti-inflammatory, both in vitro [15] and in human studies [16], which may play an essential role in post-operative analgesia.

The aim of this retrospective study is to evaluate the analgesic effects (pain scores at rest and during coughing and daily, accumulative and total opioid consumption) of intraoperative use of propofol TIVA in liver surgery. The data from patients receiving TIVA with propofol were matched with those receiving the inhalational anesthetic sevoflurane. Tolerability (side effects) and patients’ satisfaction with pain relief were also assessed.

## Methods

The study was approved by the Institutional Review Board of Queen Mary Hospital and the University of Hong Kong and registered at ClinicalTrials.gov (NCT02179437). As this was a retrospective study, there was no requirement to obtain written informed consent from patients. The data was delinked from patient identifiers and anonymized prior to analysis so that none of the researchers were aware of patient identification. Records of patients after liver surgery and under the care of the acute pain service (APS) between 1 January 2010 and 31 December 2013 in our tertiary university hospital were reviewed and analyzed. Data collected included demographic data (age, body weight, gender and American Society of Anesthesiologists (ASA) physical status); types of liver surgery performed (left or right hepatectomy, segmentectomy and wedge resection were considered as hepatectomy and radio-frequency ablation or microwave ablation were considered as RFA); types of general anesthesia techniques (total intravenous anaesthesia with propofol, inhalational anaesthesia with sevoflurane); with or without intraoperative use of ketamine; pain intensity as verbal numerical rating scale (NRS, 0 = no pain, 10 = the worst imaginable pain) at rest and during coughing from postoperative days 1 to 3; daily, accumulative and total postoperative morphine consumption and duration of PCA use; incidence of adverse events and patients’ satisfaction with pain relief. Exclusion criteria were missing essential data, difficulty in assessment of postoperative pain (e.g. postoperative mechanical ventilation, language barriers), early termination of PCA due to deterioration of patients’ condition, patients requiring a second operation and patients participating in other research projects.

None of the patients in this study received premedication. Patients in the TIVA group had anesthesia induced and maintained with propofol. For patients in the SEVO group, general anesthesia was induced with propofol 1–2 mg/kg and then maintained with sevoflurane in air and oxygen, titrated according to heart rate and blood pressure by the attending anesthesiologist. Cis-atracurium 0.15 mg/kg or rocuronium 0.6 mg/kg was administered to facilitate tracheal intubation. All patients were given a bolus of remifentanil 1mcg/kg before intubation and then an infusion titrated to hemodynamic response and maintained up to 0.2 mcg/kg/min during surgery. Morphine 0.1 mg/kg was given intravenously before incision. Additional morphine 0.1 mg/kg was given in divided doses at the discretion of the attending anesthesiologist if surgery continued for more than 2 hours. Ketamine 0.5–1 mg/kg was given at the discretion of the attending anesthesiologist. Wound infiltration with 0.5% levobupivacaine (up to 2 mg/kg) was performed by the surgeon during wound closure. After completion of surgical procedures, reversal of neuromuscular blockade was achieved with atropine 0.02 mg/kg and neostigmine 0.05 mg/kg, followed by tracheal extubation.

In the post anesthesia care unit (PACU), vital signs including blood pressure, oxygen saturation (SpO_2_) and heart rate were monitored. A 2 mg bolus of morphine was given intravenously every five minutes until the patient’s verbal numerical rating scale (NRS) was three or below. A PCA device (Graseby 3300 Syringe Pump, Smiths Medical, London), configured to give morphine at 1 mg per bolus with a five-minute lockout duration and a maximum dose limit of 0.1 to 0.15 mg/kg/hour, with the continuous basal infusion mechanism disabled, was connected for use. Subcutaneous or intramuscular morphine injection (0.05 mg/kg) was prescribed as rescue pain medication. Anti-emetics were not routinely administered for prophylaxis of postoperative nausea and vomiting (PONV), although intravenous ondansetron 4 mg 6 hourly was prescribed routinely, to be given if required.

Oral fluid intake started on postoperative day 1 if no surgical complications were apparent. Regular oral analgesics including tramadol, COX-2 inhibitors and/or paracetamol, were then started. The APS team were informed if pain control was inadequate and hourly limit and bolus dose parameters could then be adjusted after assessment. All the patients were visited daily by anesthesiologists from the APS team and postoperative pain scores at rest and during coughing, daily PCA morphine consumption and side effects were recorded until discharge from the APS. Criteria for cessation of PCA use included NRS of less than 3 during coughing, daily morphine consumption less than 0.1 mg/kg, or patients’ request. Rescue pain medication and adjuvant oral pain medication continued after cessation of PCA use. Patients were asked by the APS team to grade their satisfaction regarding the pain service as ‘good’, ‘fair’ or ‘unsatisfactory’ at the time of PCA discontinuation. They were also asked for reason(s) if they reported an ‘unsatisfactory’ pain service.

A retrospective power calculation was performed for this study. Using NRS during coughing on day 1 as the primary outcome, the mean (SD) of the TIVA group was found to be 4.301 (1.986), while the mean of the SEVO group was 5.08, at 80% power and an alpha of 0.05, 52 subjects would be required in each group.

Each patient who received TIVA was matched 1:1 to patients who had received sevoflurane according to age, gender, ASA physical status, types of liver surgery performed (hepatectomy vs radio-frequency ablation) and with or without intraoperative use of ketamine [17]. This one to one patient matching is a more comprehensive method than using the propensity score [18, 19]. Patient demographics are presented as means (SD) for parametric data, and percentage where appropriate. Differences in pain scores were tested for using unpaired t-test with Welch’s correction, while Mann-Whitney test was used to look at differences in morphine use. Differences in post-operative side effects and overall satisfaction was tested for using Fisher’s Exact test. Statistical Package for the Social Sciences (SPSS Statistics version 20, IBM Corp., USA) statistical software was used for data analysis.

## Results

One thousand two hundred and eighty two records of patients who underwent liver surgery under the management of the APS team were screened. It was found that 95 patients had received TIVA with propofol as maintenance of general anaesthesia during the specified period, while 720 patients received sevoflurane. Patient demographics are shown in Table 1. No statistically significant differences were seen between the two groups in either the duration of surgery or the use of intraoperative opioid.

**Table 1:** Patient demographics, duration of surgery and total intraoperative opioid use. Values in means ± SD (range) or %.

	SEVO(n = 95)	TIVA(n = 95)
Age (years)	60.24 ± 10.40(31–83)	60.72 ± 10.33(32–89)
Body weight (kg)	63.71 ± 12.15(45–100)	61.66 ± 11.64(34–102)
Sex, M: F	73:23	73:23
ASA, I: II: III	5:63:27	3:61:31
Surgery type, RFA: Hepatectomy	23:77	23:77
Ketamine: no ketamine use	82:18	89:11
Duration of surgery (mins)	368.7 ± 206.3(74.0–1235)	357.6 ± 194.6(119.0–1079)
Total intraoperative opioid use	267.5 ± 190.4(0.0–925.0)	326.4 ± 459.9(0.0–3959)

Pain scores as NRS at rest and during coughing for the first three postoperative days are presented in Table 2. There were no significant differences in NRS pain score at rest between the two groups. The TIVA group reported a lower NRS pain score on coughing with a mean of 4.301 and 4.000 on postoperative days 1 and 2 respectively (p<0.05), but no differences between the groups was seen on day 3.

**Table 2:** Postoperative pain scores. Values in means (SD).

	SEVO(n = 95)	TIVA(n = 95)	*p* value
**NRS Pain scores at rest**			
Day 1	2.159 (1.825)	1.771 (1.655)	0.1468
Day 2	1.307 (1.307)	1.376 (1.456)	0.7412
Day 3	1.100 (1.199)	1.180 (1.466)	0.7659
**NRS Pain scores during coughing**			
Day 1	5.080 (2.052)	4.301 (1.986)	0.0127
Day 2	4.563 (1.796)	4.000 (1.896)	0.0472
Day 3	4.380 (1.872)	4.120 (1.586)	0.4556

NRS = Numerical Rating Scale

Daily morphine consumption is shown in Table 3. There was a reduced daily mean morphine consumption in the TIVA group on postoperative days 1 to 3 (8 mg vs 12 mg, p = 0.001 on day 1, 9.0 mg vs 13.0 mg, p = 0.0231 on day 2 and 16.0 mg vs 28.0 mg on day 3, p = 0.0004). Total PCA morphine consumption was also less in the TIVA group when compared with the SEVO group (p = 0.03). However, there was no difference in duration of PCA use (59 hours in both groups, Table 4).

**Table 3:** Daily Morphine consumption (mg) from postoperative day 1 to 3. Values in median, [range] (mean).

	SEVO	TIVA	P value
Day 1	12.0[1–61.0](15.76)	8.0[0–55.0](10.21)	0.001
Day 2	13.0[1.00–74.0](16.50)	9.0[0–81.0](13.55)	0.0231
Day 3	28.0[7.0–85.0](31.51)	16.0[1–61.0](18.74)	0.0004

**Table 4:** Total post-operative morphine consumption (mg) and duration of PCA use. Values in median [IQR] (range).

	SEVO(n = 95)	TIVA(n = 95)	P value
Total PCA morphine consumption (mg)	30 [18–58.25](1–154)	21.5 [9.75–40.25](1–453)	0.03
PCA duration (hours)	59 [43.75–70.5](20–114)	59 [43–68](14–136)	0.9857

No difference was seen between the number of patients suffering from nausea, dizziness and pruritus in either group, and only the incidence of pruritus was lower in the TIVA group (p = 0.029). Since side effects were a rare occurrence in both treatment groups, and though statistical analyses and comparisons were performed, the low rate of events renders the power to detect differences in side effects to be very low (Table 5). Seventy five point eight percent of patients in the TIVA group and 77.9% of patients in the SEVO group graded overall satisfaction as ‘fair’, and no differences in overall satisfaction was seen (Table 6).

**Table 5:** Postoperative side effects. Values in n (%).

	SEVO(n = 95)	TIVA (n = 95)	P value
Nausea	15 (15.8%)	11 (11.6%)	0.527
Vomiting	6 (6.3%)	6 (6.3%)	1.00
Dizziness	5 (5.3%)	1 (1.1%)	0.211
Pruritus	6 (6.3%)	0 (0%)	0.029
Postoperativedelirium	0	0	1.00

**Table 6:** Overall satisfaction with postoperative pain control. Values in n (%).

	SEVO(n = 95)	TIVA(n = 95)	P value
Overall satisfaction			
Good	7 (7.4%)	7 (7.4%)	1.00
Fair	74 (77.9%)	72 (75.8%)	0.86
Unsatisfactory	0 (%)	0 (%)	1.00
Unknown	14 (14.7%)	16 (16.8%)	0.84

## Discussion

Our study showed that patients receiving propofol for maintenance of general anaesthesia in liver surgery reported significantly less pain as reflected by lower mean NRS pain scores during coughing and less daily, accumulative and total postoperative morphine consumption. With a large upper abdominal wound, high doses of strong opioids may be required for pain control but its side effects may become apparent as morphine metabolism is impaired after liver resection, especially for those with liver cirrhosis and impaired liver function [3]. There are also restrictions in analgesic selection. Paracetamol is not routinely prescribed for fear of drug induced hepatotoxicity in patients who have undergone hepatectomy and, if prescribed, the dose should be limited to 2–3 g/day [1, 20]. In view of possible thrombocytopenia secondary to hypersplenism, deranged clotting profile, potential major blood loss, and renal impairment from the hepatorenal syndrome, non-steroid anti-inflammatory drugs (NSAID) are relatively contraindicated [1, 20]. Although epidural analgesia using morphine and ketamine [21], bupivacaine and fentanyl [22], or a single bolus of preoperative intrathecal morphine [23] have been shown to provide reasonable pain relief after liver resection surgery, those patient groups had predominantly undergone liver resection for colorectal metastasis [22] or were healthy liver donors for right hepatectomy [23]. The complication of epidural hematoma is devastating and patients with cirrhosis, thromobocytopenia and post liver resection coagulopathy are at particular risk, such that most anesthesiologists will avoid epidural analgesia in this setting. It is important to achieve good acute pain control to prevent progression to chronic pain and facilitate early mobilization. It has been shown that higher postoperative morphine consumption at 24 hours is predictive of the development and severity of chronic pain [24]. Therefore, post-operative pain control in this group of patients can be a major challenge [1]. Propofol was primarily developed as an anesthetic and sedative drug and the potential analgesic effect is an interesting and serendipitous discovery [25]. With regard to acute pain, a study by Cheng and colleagues showed that propofol was associated with less postoperative pain and less self-administered morphine when compared with isoflurane in the first day after open uterine surgery [25]. Two further studies found that patients who underwent laparoscopic gynecological surgery under propofol anesthesia reported less pain over the immediate postoperative period compared with those receiving sevoflurane. In the study where Li and colleagues studied 90 patients, the pain scores at rest at 0.5 hour and 1 hour postoperatively were significantly lower in the propofol group compared with the sevoflurane group [26]. Tan et al found that the pain scores were significantly higher in the sevoflurane group when compared with the propofol group over the immediate postoperative 4 hour period [27]. In a study of pediatric patients undergoing hernia repair, propofol use was found to be associated with a lower proportion of patients exhibiting postoperative pain when compared with sevoflurane [28]. On the contrary, a study comparing sevoflurane and propofol found no difference in cumulative opioid consumption and pain scores at rest or after cough at postoperative 2, 4, 8 and 24 hours after abdominal hysterectomy or myomectomy [29]. Anesthetic techniques as propofol TIVA are potentially useful in decreasing postoperative pain and opioid consumption, especially in fast track surgery [4] and should be considered an option for reducing postoperative pain.

In contrast to previously mentioned studies which measured pain scores at rest [26], our study demonstrated a reduction in pain scores during coughing which is important in chest physiotherapy and mobilization, especially with large upper abdominal wounds. In addition, compared with other studies which measured pain scores from 0.5 to 24 hours postoperatively [25–27], our study showed that the decrease in pain scores extended to postoperative day 2, with less daily morphine consumption up to day 3. This makes it very unlikely that the reduction in pain scores and morphine consumption within the first 24 hours is due to a sedative effect from residual anesthetics because propofol has a very fast recovery profile. Although there was no decrease in pain scores at rest and total duration of PCA use, the decrease in total morphine consumption by 28% in our study is both statistically and clinically significant.

As well as improving patient comfort, adequate relief of pain on movement and during coughing is important in reducing risks of cardiopulmonary and thromboembolic complications after surgery [30]. In addition, immobilization after surgery is a known risk factor for the development of chronic hyperalgesia [31], affecting around 1% of surgical patients. Effective relief of pain on movement post-surgery is, therefore, important to improve long-term outcome [30, 31].

Propofol’s exact mechanism of action remains unknown, though both cell culture and animal studies suggest that it is through interaction with GABA_A_ receptors that propofol exerts its anesthetic as well as its analgesic effects [14, 25, 32, 33]. Animal studies employing the delta opioid antagonist naltrindole suggests that as well as working through the GABA_A_ receptors, propofol antinociception is also mediated by a spinal delta opioid receptor [32]. Another potential route through which propofol exerts its analgesic effect [34] is through its anti-inflammatory [35] and antioxidant action [36].

With regard to side effect profiles, our results showed no difference in the incidence of nausea, vomiting, dizziness, or post-operative delirium between the SEVO and the TIVA groups, although pruritus was significantly lower in the TIVA group. It is to be noted here that this study was not powered to detect side effects with a low incidence and, essentially, raw data does show that 15.8% of the SEVO group had nausea, versus 11.6% in the TIVA group, while 5.3% in the SEVO group reported dizziness, versus 1.1% in the TIVA group.

There are several limitations to this study. Firstly, this was retrospective with data retrieved from a registry. We did not utilize a standardized target for maintenance of depth of general anesthesia such as processed EEG. Another limitation is the interplay between drugs and hepatic function. There is uncertainty about the remaining liver function after hepatectomy compared with radiofrequency ablation, although studies have shown that patients who received radiofrequency ablation exhibited less derangement in albumin and bilirubin levels on postoperative day 7 [37]. In addition, it is unclear how hepatic function affects the metabolism of sevoflurane, propofol and morphine. With regard to propofol, studies have shown no requirement to change the infusion dose in patients with moderate cirrhosis [38, 39]. Sevoflurane, being an inhalational anesthetic with low blood:gas solubility undergoes only 2–5% metabolism by the liver [40]. Although we did not measure postoperative sedation, the possibility that patients may become more sedated and hence consume less morphine because of residual anesthetic was unlikely as our result showed the decrease in NRS pain scores during coughing was still apparent at postoperative day 2 and daily morphine consumption was also lower, outlasting the therapeutic duration of both sevoflurane and propofol.

In conclusion, patients who underwent liver surgery using propofol for induction and maintenance of anesthesia had less pain during coughing on postoperative days 1 and 2 and reduced daily, accumulative and total postoperative morphine consumption when compared to the use of sevoflurane. No difference was found in pain at rest on post-operative day 1 to 3, total duration of PCA use and patients’ satisfaction with pain relief. The incidence of post-operative nausea, dizziness and pruritus was lower in the TIVA group.
